# Cystic pancreatic lymphangioma: a case report

**DOI:** 10.1186/s13256-022-03730-y

**Published:** 2023-01-24

**Authors:** Hiwote Girma Assefa, Tafese Gudissa Merga, Binyam Gebremedhin Godu

**Affiliations:** Saint Paul’s Hospital Millennium Medical College, Addis Ababa, Ethiopia

**Keywords:** Pancreatic lymphangioma, Vascular tumors

## Abstract

**Background:**

Lymphangiomas are benign vascular tumors arising from the lymphatic system. They commonly affect the head and neck regions. Pancreatic involvement is extremely rare. Even though they are rare, it should be considered as a differential diagnosis for patients who present with pancreatic mass.

**Case presentation:**

We report the case of a 6-year-old African male patient who presented with abdominal mass and dull aching pain of 6 months duration. He was examined and underwent excision, with biopsy showing pancreatic lymphangioma.

**Conclusion:**

These tumors are benign and slow growing and have potential to regress spontaneously. Thus, most literature agrees that surgical interventions should be reserved for symptomatic cases.

## Introduction

Lymphangiomas are benign congenital tumors of lymphatic vascular origin. They commonly affect head and neck regions. Pancreatic involvement is extremely rare. These tumors are usually congenital but can arise as a result of blockage of lymphatic flow from trauma, fibrosis, inflammation, or radiotherapy. Patients are usually asymptomatic but can present with abdominal pain and mass. We report the case of pancreatic lymphangioma in a 6-year-old male patient presenting with abdominal pain and mass.

## Case presentation

**Fig. 1:**
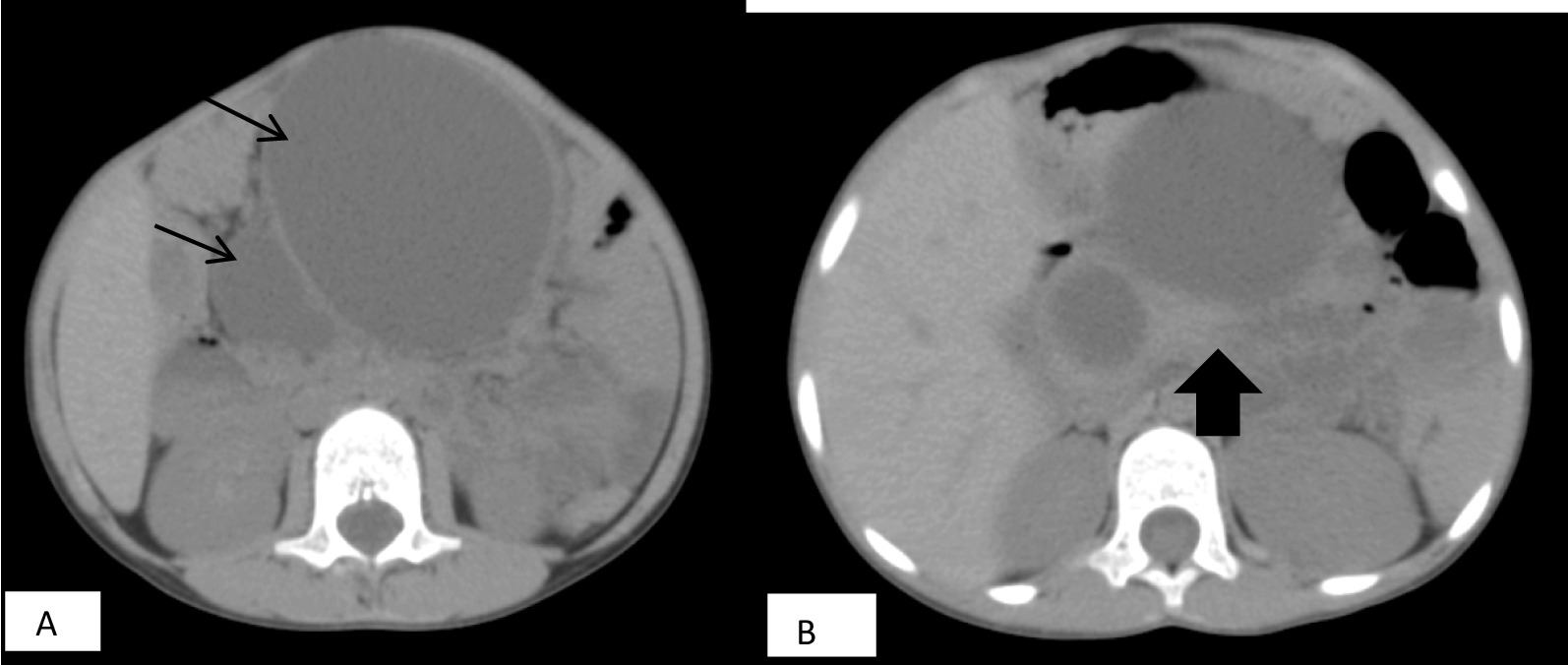
Computed tomography scan of pancreatic lymphangioma. **A** Axial view both upper and lower arrows showing different size non-communicating multicystic retroperitoneal mass. **B** As depicted by the arrow, no clear plane is seen between the mass and the pancreatic tissue

A 6-year-old African male child presented with abdominal mass and dull aching type of abdominal pain of 6 months duration that worsened 1 month before presentation. He had no past medical illness or surgical interventions. He also had no family history of similar illness. Upon our evaluation, the patient was stable. There was an ill-defined, smooth-surface, nontender, fixed, intra-abdominal mass at the center of the abdomen extending to the left upper quadrant measuring about 8 cm × 10 cm. Liver function test, renal function test, pancreatic enzymes, and tumor markers were all in normal range. Abdominal computed tomography (CT) scan showed multiple cystic mass adjacent to each other with no communication between the masses; the largest cyst measured about 8 cm × 7.5 cm × 9.5 cm. The mass was found adjacent to the head of the pancreas with no clear plane between the mass and pancreas (Fig. 1). It was difficult to reach a conclusive diagnosis from the CT scan as there were multiple diagnoses presented to us by the radiologist, including pseudocyst and duodenal duplication, but the senior radiologist raised the possibility of pancreatic lymphangioma. Since the patient had severe abdominal pain, we opted to explore this patient despite diagnostic dilemma.

**Fig. 2:**
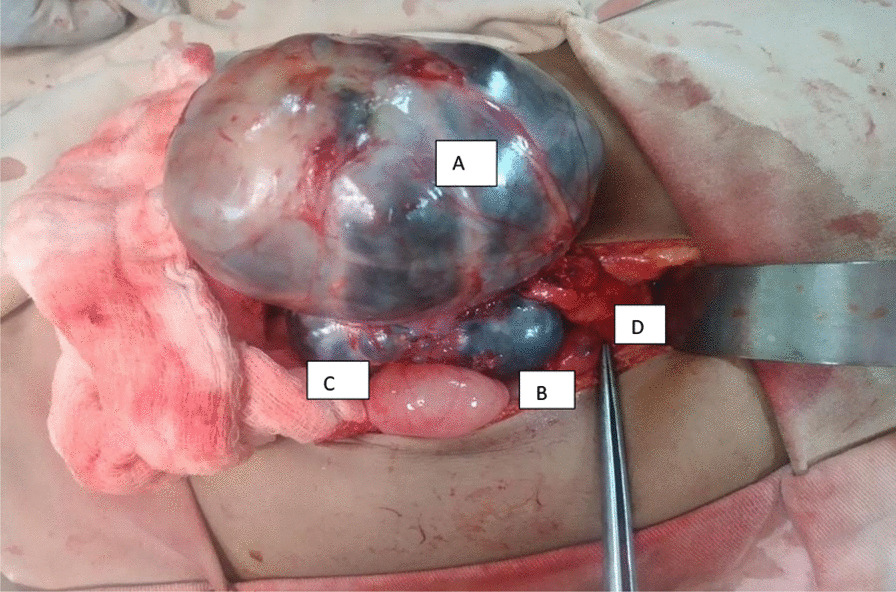
Intraoperative findings of pancreatic lymphangioma. **A**–**C** Multiloculated cystic retroperitoneal mass engulfed by the pancreas. **D** Part of head of pancreas

The patient was operated, with an intraoperative finding of well-capsulated multiloculated cystic mass that was wrapped with pancreatic tissue and no sign of local invasion (Fig. 2). The mass had no invasion to major structures, and so it was gently mobilized from the pancreatic bed, completely excised without spillage, and sent for histopathology evaluation; The result confirms cystic lymphangioma. The patient was discharged improved and is currently on follow-up with smooth postoperative course, and there are no signs of recurrence.

## Discussion

Lymphangiomas are benign vascular tumors arising from the lymphatic system [1]. They account for about 5% of tumors in children and are commonly found in the head and neck region [2]. Other sites reported in the literature include pleura, pericardium, groin, bones, liver, spleen, pancreas, colon, omentum, and genital organ [4].

Pancreatic lymphangioma is a very rare tumor accounting for less than 1% of abdominal lymphangioma and less than 0.5% of all cystic pancreatic lesion [3]. These tumors result from lymphangiectasia as a consequence of a blockage of lymphatic flow. This may be associated with congenital malformations or obstructions as a result of an inflammatory process, radiotherapy, surgery, or any abdominal trauma [3]. It is more common in the pediatrics population, which is similar in our case and has a slight female preponderance [1].

These tumor are benign in nature, slowly growing, and usually asymptomatic [6]. Some patients present with abdominal pain and associated abdominal mass [6–8], as does our patient.

There are no associated laboratory findings specific to this abnormality. Abdominal imaging such as ultrasound and CT scan assist in the diagnosis and evaluation of such lesions; however, imaging by itself is not 100% specific to differentiate lymphangiomas from other cystic pancreatic lesions such as pseudocysts, cystadenomas, congenital cysts, and ductal carcinomas. Thus, this pathology can be differentiated from other cystic pancreatic lesions by histopathologic examination [3, 9, 10]. On abdominal ultrasound, the typical presentation of lymphangioma is multilocular lesion with homogeneous serous composition that appears anechoic. CT scan revealed a multiloculated cystic mass with fluid attenuation, thin septa within the lesion, and nonenhancing with no solid component or calcification seen. The tumor does not invade adjacent structures, which was similar to our patient’s CT scan.

Pathologically, lymphangioma can be classified as macrocystic, microcystic, or mixed-type lymphangioma. Macrocystic lymphangiomas are greater than 1 cm in diameter and occur in areas with loose connective tissue such as in the abdomen, neck, and axilla, while microcystic lymphangiomas are less than 1 cm in diameter and arise in areas with dense connective tissue such as tongue and lip. The natural history of lymphangioma depends on its histology type. Macrocystic and mixed types can spontaneously regress, while microcystic lymphangioma does not regress. Therefore macrocystic and mixed types can be serially followed in asymptomatic patients with stable tumor [6, 10].

Surgical interventions are reserved for patients with symptomatic pancreatic lymphangioma and for those with diagnostic dilemmas. Excision of the mass is considered to be curative. As there is a high chance of recurrence, the mass should be removed in its entirety [2, 3, 5]. We have also removed the mass as a whole in our case. Some patients might also require extensive pancreatic resections such as Whipple procedure or distal pancreatectomy depending on the size and location of the mass [3].

## Conclusion

Pancreatic lymphangioma, even though it is a rare tumor, should be considered as a differential diagnosis in patients who present with cystic pancreatic mass. This tumor is benign and slow growing and has the potential to regress. Thus, surgical interventions should be reserved for symptomatic cases.

## Data Availability

All data and materials are available upon request by the Editor-in-Chief.
